# Low-temperature microstructural studies on superconducting CaFe_2_As_2_

**DOI:** 10.1038/s41598-019-42660-6

**Published:** 2019-04-23

**Authors:** S. Huyan, L. Z. Deng, Z. Wu, K. Zhao, J. Y. Sun, L. J. Wu, Y. Y. Zhao, H. M. Yuan, M. Gooch, B. Lv, Y. Zhu, S. Chen, C. W. Chu

**Affiliations:** 1 Department of Physics and Texas Center for Superconductivity, University of Houston, Houston, TX 77204-5005 USA; 2 Condensed Matter Physics and Materials Science Department, Brookhaven National Laboratory, Upton, NY 11973 USA; 3 School of Physics and Optoelectronic Engineering, Nanjing University of Information Science and Technology, Nanjing, China; 4 Department of Physics, University of Texas at Dallas, Richardson, TX 75080 USA; 5 Lawrence Berkeley National Laboratory, Berkeley, CA 94720 USA

**Keywords:** Superconducting properties and materials, Superconducting properties and materials

## Abstract

Undoped CaFe_2_As_2_ (Ca122) can be stabilized in two slightly different non-superconducting tetragonal phases, PI and PII, through thermal treatments. Upon proper annealing, superconductivity with a T_c_ up to 25 K emerges in the samples with an admixture of PI and PII phases. Systematic low-temperature X-ray diffraction studies were conducted on undoped Ca122 samples annealed at 350 °C over different time periods. In addition to the diffraction peaks associated with the single-phase aggregation of PI and PII, a broad intermediate peak that shifts with annealing time was observed in the superconducting samples only. Our simulation of phase distribution suggests that the extra peak is associated with the admixture of PI and PII on the nanometer scale. High-resolution transmission electron microscopy confirms the existence of these nano-scale phase admixtures in the superconducting samples. These experimental results and simulation analyses lend further support for our conclusion that interfacial inducement is the most reasonable explanation for the emergence of superconductivity in undoped Ca122 single crystals.

## Introduction

Possible interface-enhanced superconductivity was proposed more than 50 years ago^1^. In the ensuing years, many experiments have been carried out and numerous reports of enhanced T_c_ have appeared^2^. For example, induced or enhanced T_c_ has been reported in artificially fabricated bilayer structures of different materials, such as LaAlO_3_/SrTiO_3_ with T_c_ ~ 0.2 K^3–7^, where both starting compounds LaAlO_3_ and SrTiO_3_ are non-superconducting; La_1.55_Sr_0.45_CuO_4_/La_2_CuO_4_ with T_c_ ~ 50 K^8^, where, likewise, neither of the starting compounds is superconducting; and unit-cell-layer FeSe/SrTiO_3_ with an enhanced T_c_ above 45 K^9–11^, where bulk FeSe has a T_c_ of only ~8 K. These observations have been attributed to exotic excitons^12^ or to conventional soft phonons at the interfaces^13,14^. It should be noted that complexities often exist in thin-film processing, arising from stress/strain and material diffusion near the interfaces.

Recently, non-bulk superconductivity up to 49 K was discovered in rare-earth-doped CaFe_2_As_2_ (Ca122) at ambient pressure^15–17^. Ensuing studies suggested that the superconductivity might be related to interfaces associated with defects in the samples^18,19^. However, the evidence for possible interface-induced superconductivity is not clear in this case due to the complications involved in doping. Therefore, undoped Ca122, without the complexities described above, would be an ideal system in which to seek clear direct evidence. The undoped Ca122 displays a complex temperature-pressure phase diagram with superconductivity appearing only in some of the phases and only under non-hydrostatic pressures^20,21^. One of the most intriguing observations has been the filamentary superconductivity up to 10 K detected sporadically under ambient pressure^22^. Although the related shielding volume fraction is much higher under non-hydrostatic pressure, the complex pressure environment makes it difficult to delineate the relationship between different phases and superconductivity^20,21^. Later studies have shown that the complex phase evolution under pressure can be reproduced at ambient pressure by proper heat treatment^23–25^.

It was later found^23–28^ that the undoped single-crystalline Ca122 can stabilize in two slightly different non-superconducting tetragonal phases (PI and PII) after rapid quenching from 850 °C and prolonged annealing at 350 °C, respectively. The PI phase corresponds to a tetragonal (T) structure at room temperature [lattice parameter *c* = 11.547(1) Å], and transforms to a collapsed-tetragonal (cT) phase with a 10% shorter *c*-lattice [*c* = 10.720(1) Å] below the T-cT structural transition temperature (T_cT_) around 100 K. On the other hand, the PII phase also exhibits a tetragonal structure at room temperature but with a slightly longer *c* [*c* = 11.763(1) Å]. It undergoes a tetragonal-to-orthorhombic (T-O) transition [*c* = 11.653(1) Å] at the T-O structural transition temperature (T_O_) around 170 K. The orthorhombic phase is ordered antiferromagnetically, and the T-O transition is closely related to the spin-density-wave transition. It was found that the PI and PII phases could be reversibly transformed by heat treatment^28^. Microscopic techniques were also utilized to reveal the homogeneity and strain effect in the Ca122 annealed at different temperatures^23,24^.

Interestingly, the Ca122 sample with a pure-PI phase will undergo a transition from the pure-PI phase, to a PI + PII-mixing phase, and finally to the pure-PII phase upon annealing at a low temperature, i.e. 350 °C, over increasing time periods of 0–30 hours. It was found that as PI is gradually converted to PII, superconductivity at ~25 K emerges over a limited annealing time window^28,29^. The simultaneous appearance of the PI + PII-mixing phase and superconductivity at ambient pressure enables us to examine the microscopic relationship between the coexistence of the mixing phase and superconductivity.

To understand the occurrence of superconductivity in Ca122 with an admixture of PI and PII phases, the mesoscopic structures, especially the interfacial structures, need to be clarified. We have therefore taken advantage of the high-quality X-ray diffraction (XRD) signals and performed a series of XRD studies at low temperature on Ca122 single-crystalline samples annealed for different time periods at 350 °C and then quenched to room temperature in ice water. In addition, high-resolution transmission electron microscopy (HRTEM) has been employed to elucidate the mixed structure at the atomic level. The XRD results indicate that the observed superconductivity is the result of the thorough mixing of the nanoscale PI and PII phases. The HRTEM data also show the admixture of PI and PII domains of <10 nm in our superconducting samples. These results provide the clearest and strongest evidence to date for interface-induced superconductivity with T_c_ up to 25 K in undoped Ca122 single crystals.

## Results

**Figure 1:**
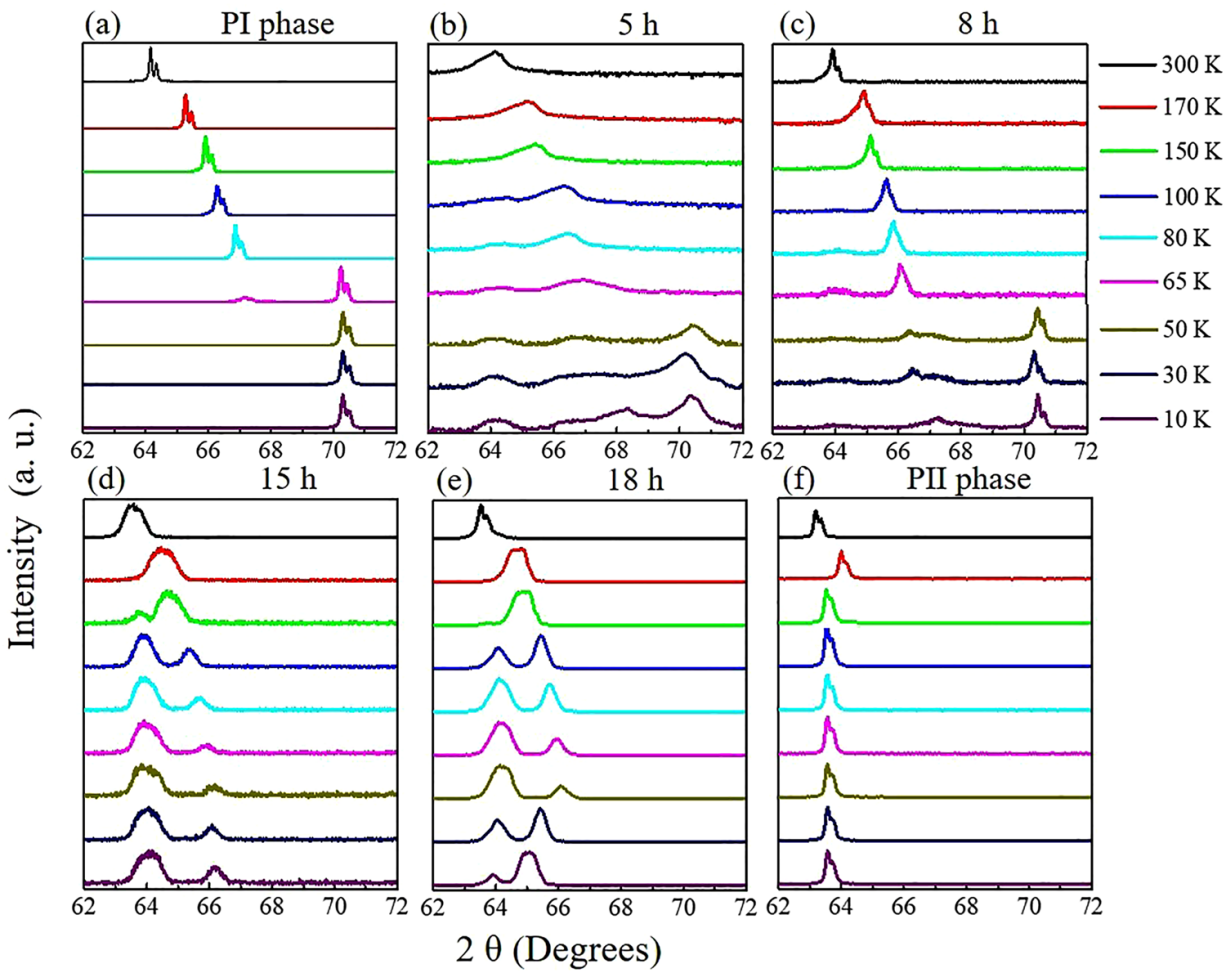
Low-temperature XRD focused on the single-crystal (008) peak of undoped CaFe_2_As_2_. (**a**) Pure PI phase. Starting from the PI phase, the sample is annealed at 350 °C for (**b**) 5 h (intensity in log scale), (**c**) 8 h, (**d**) 15 h, (**e**) 18 h, and **(f**) 30 h (completely transformed into pure PII phase).

We have examined the XRD spectra of non-superconducting samples of pure-PI and -PII phases. Both phases have a tetragonal structure at room temperature but with slightly different c-lattices, i.e. 11.55 Å and 11.76 Å for PI and PII, respectively. On cooling, the PI phase sample undergoes a tetragonal to collapsed-tetragonal structural transition (T-cT transition) below ~70 K, with a 10% shorter c-lattice constant. This is evidenced by the rapid higher-angle shift of the (008) peak position in the XRD spectra at ~70 K. As shown in Fig. 1(a), the (008) peak appears at ~64° at 300 K. On cooling to 80 K, the (008) peak moves gradually to increasingly larger angles associated with thermal contraction. Below 65 K, the peak suddenly moves several degrees to around 70.3°, corresponding to the collapse of the *c*-axis associated with the T-cT transition. The PII-phase sample also has a tetragonal structure, but with a slightly larger *c*-lattice constant at room temperature as shown in Fig. 1(f). Upon cooling to below 170 K, the sample transforms to an orthorhombic structure (T-O transition). The XRD spectra in Fig. 1(f) show that the (008) peak at ~62.6° at 300 K moves to ~64.2° gradually during cooling, and then goes back to 63.7° below 150 K, corresponding to the T-O transition. According to Fig. 1(a,f), the positions of the (008) peaks shift negligibly below the respective PI and PII structural transition temperatures when only the PI or the PII phase exists. For these samples, no other diffraction peaks were observed in the 2θ range of 62°–72°. The twin peaks in the Fig. 1 spectra are due to the Cu-K-alpha doublet.

We then investigated a series of samples that consist of an admixture of the two phases (PI + PII). The samples, all starting from the pure PI phase, were annealed at 350 °C for 5 h, 8 h, 15 h, and 18 h, respectively, and then quenched in ice water. Superconductivity was observed in all four samples electrically as well as magnetically (Fig. S1). The low-temperature XRD results are displayed in Fig. 1(b–e). For the sample annealed for 5 h, shown in Fig. 1(b) (the intensity of diffraction peaks is displayed in log scale), the PI and PII phases coexist. On cooling the sample from 300 K, it is observed that the (008) peak shifts to a larger angle. Below 150 K, a very small bump starts to appear at around 64°, indicating a T-O transition of the PII phase. The very low intensity of the bump indicates a small amount of the PII phase in the sample. At 50 K, a much stronger peak at around 70.3° is observed, corresponding to the existence of the dominant PI phase in the sample. In addition, an extra intermediate small bump at ~68.4° is observed below the T-cT transition temperature. For the samples annealed for 8 h, shown in Fig. 1(c), the 64° peak is clearly observed in linear scale, which indicates a larger portion of the PII phase as expected. Meanwhile, the intermediate small bump is shifted to a lower angle as compared with that of the sample annealed for 5 h. For the sample annealed for 15 h, shown in Fig. 1(d), the PII phase is dominant, as demonstrated by the XRD peaks, where a majority of the peaks belong to the PII phase. The intermediate small bump is also further shifted to a lower angle. The same trend is also observed in the sample annealed for 18 h as shown in Fig. 1(e). We interpret the XRD data for the different samples as follows: **(1)** two diffraction peaks at around 70.3° and 64°, observed below 70 K and 170 K, respectively, indicate the single-phase aggregation of PI and PII, respectively; **(2)** the extra intermediate peak indicates the nano-scale phase admixture of PI and PII; and **(3)** the difference in the intermediate peak position with annealing time is caused by the change in the PI/PII phase ratio.

**Figure 2:**
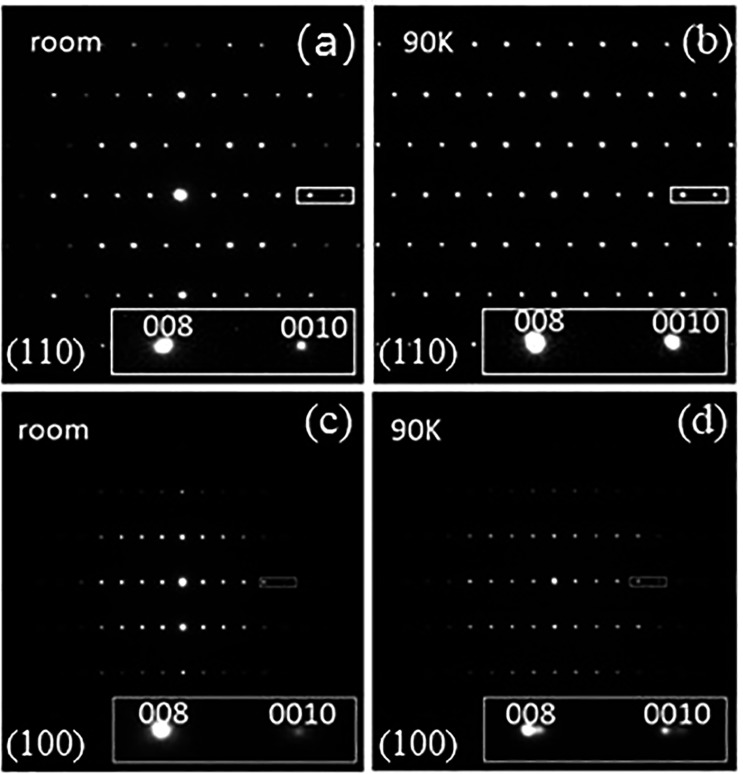
SAED patterns of a PII sample at (**a**) room temperature and (**b**) 90 K; and of a 15-h-annealed sample at (**c**) room temperature and (**d**) 90 K. Insets are the magnified images of (008) and (0010) peaks.

To further identify the phase distribution in superconducting Ca122, HRTEM experiments on both the pure PII-phase sample and the superconducting sample annealed for 15 h were conducted. For effective comparison with the low-temperature XRD results, we have examined the samples both at room temperature and at 90 K. The selected-area electron diffraction (SAED) patterns of these two samples at room temperature and 90 K are shown in Fig. 2. No spot splitting can be detected in the diffraction pattern of the PII-phase sample when the temperature decreases from room temperature to 90 K [Fig. 2(a,b) and insets]. For the mixed-phase sample, on the other hand, the (008) spot splits into two at 90 K, as does the (0010) spot [Fig. 2(c,d) and insets], resulting from a 2.02% difference in the *c* value at 90 K. This is very close to our XRD results on the 15-h-annealed sample (Fig. 1(d)) at 100 K, where the peaks at 64° and at ~65.5° indicate a *c* value difference of 2.22%.

**Figure 3:**
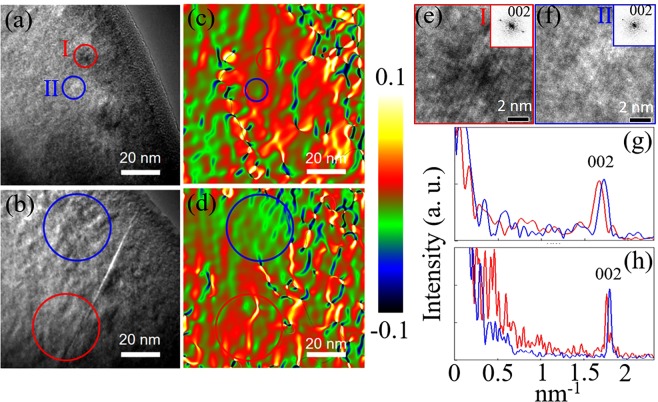
(**a,b**) HRTEM images of two typical areas of the 15-h-annealed sample and (**c,d**) their corresponding strain maps by GPA, where the colors represent different strain levels. (**e,f**) The enlarged images of areas I and II in (**a**), respectively, with the insets showing their respective electron diffraction patterns. (**g,h**) Intensity profile of the FFT patterns of the areas inside the red circles (red curves) and blue circles (blue curves) of (**a,b**), respectively.

Geometrical phase analysis (GPA) along the ε_zz_ direction was then applied to the HRTEM images (Fig. 3(a,b,e,f) are enlarged images that show clear lattice fringes. The strain maps were calculated from these lattice fringes.]. The GPA mappings (Fig. 3(c,d)) show positively- and negatively-strained domains with domain size ranging from 8 to 20 nm. Fast Fourier transforms (FFT) were also performed on the areas with positive strains, marked by red circles, and on those with negative strains, marked by blue circles. The intensity profiles (Fig. 3(g,h)) from the central spot along the (002) spot direction were then obtained from the FFT patterns. Clearly, the positions of the (002) spots of the areas inside the red circles (the red curves) are slightly closer to the central spot as compared with those of the areas inside the blue circles (the blue curves), confirming the larger *c* value of the red-circled areas. Thus, we attribute the negatively-strained domain to the low-temperature phase of PI and the positively-strained domain to that of PII. Since the domain size of the PI and PII phases can vary from a few nm to tens of nm, corresponding to the range between a few layers and tens of layers, a mixing structure formed by a few layers of each phase repeatedly stacking on one another is a reasonable explanation for the emergence of the intermediate peak observed by low-temperature XRD.

## Discussion

To test our hypothesis regarding the micro- and nano-structures of the superconducting samples, we employed a toy model to simulate the distribution of the PI and PII phases in the sample. The negative binomial distribution (NBD) model has been broadly applied in various areas, e.g. crystal growth^30^, so we have attempted to simulate the phase distribution patterns under different annealing processes using NBD and to transform the patterns into XRD spectra using the intergrowth model^31^.

**Figure 4:**
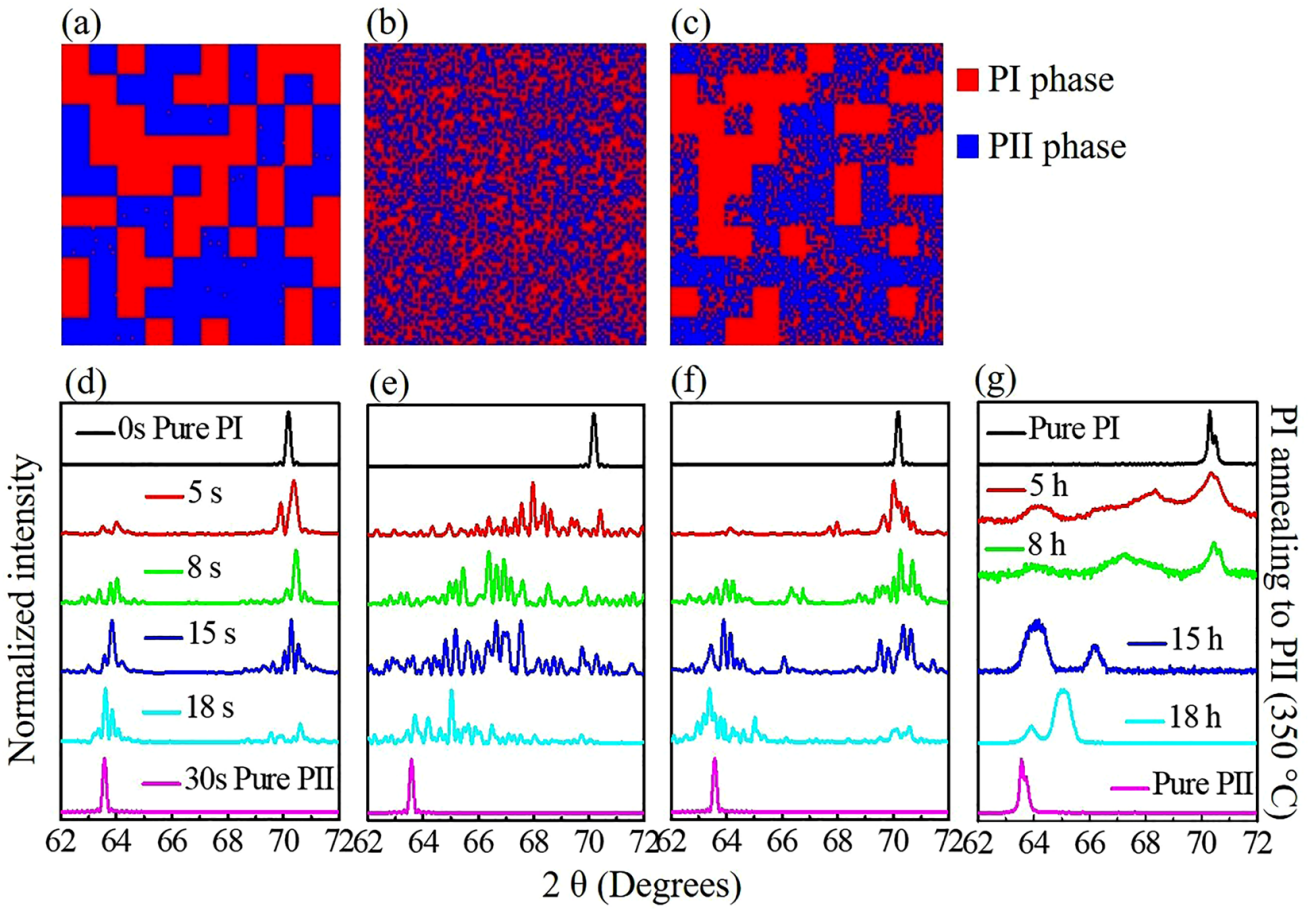
Two-dimensional simulation of phase distribution at 15 s when PI: PII = 0.5 (*p* = 0.5) and the setting aggregation parameter *r* is (**a**) 1000, (**b**) 1, and (**c**) 400. Simulated 10-K XRD spectra for samples annealed at 350 °C over different time periods with *r* of (**d**) 1000, (**e**) 1, and (**f**) 400. (**g**) Experimental data at 10 K extracted from Fig. 1 (5 h and 8 h data plotted in log scale).

Figure 4(a–c) show the simulated phase distribution in the 15-h-annealed sample. Figure 4(d–f) present the simulated results of the evolution of the XRD pattern at 10 K over different annealing time periods. Figure 4(g) provides the experimental XRD spectra of different samples at 10 K. By comparing the simulation with the experimental results, we conclude that the intermediate peaks observed by XRD in the superconducting samples may be related to the random stacking of nano-scale PI and PII phases. To simulate the phase distribution, three typical situations are presented: **(1)** Two phases in a sample exhibit full phase separation as shown in Fig. 4(a). In this case, the aggregation parameter *r* is extremely large, e.g. r = 1000. Only two characteristic peaks that belong to the PI and PII phases can be observed, as shown in Figure 4(d). **(2)** In the other extreme situation, the PI and PII phases are thoroughly mixed, as shown in Fig. 4(b). The aggregation parameter *r* in this case is very small, e.g. r = 1. As shown in Fig. 4(e), a broad peak area appears in the simulations of samples annealed for 5, 8, 15, and 18 h, rather than the characteristic peaks of the PI and PII phases. **(3)** The aggregation of the PII phase in the samples is neither too small nor too large. In this case, the corresponding aggregation parameter *r* is assigned an intermediate value (we chose r = 400), and the PI and PII phases exhibit partial large-scale pure-phase aggregation and partial phase admixture as shown in Fig. 4(c). As shown in Fig. 4(f), the characteristic peaks of the PI and PII phases can be observed, as well as an obvious extra peak between those characteristic peaks. These simulation results are consistent with our experimental results, as shown in Fig. 4(g). Therefore, we propose that the superconductivity is related to the intermediate extra peak, i.e., the interfaces between the mixed PI and PII phases.

**Figure 5:**
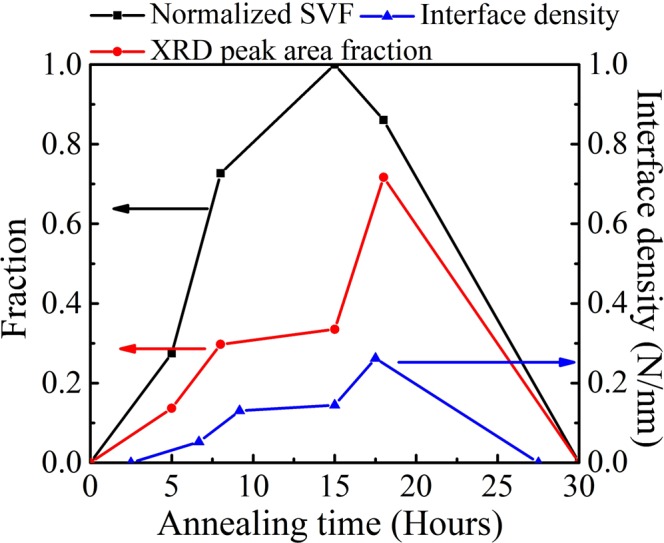
Comparison among the shielding volume fraction at 2 K, the weight of the intermediate peak area over the whole (008) peak spectrum, and the estimated interface density.

Additionally, we estimated the intermediate peak area fractions of different samples at 10 K based on Fig. 4(g). These fractions can be associated with the weight of the phase admixture in the samples as suggested by the simulation. The normalized shielding volume fraction (SVF) at 2 K values were obtained through magnetic measurements (as shown in Fig. S1), and we find that the profile of normalized SVF values with different annealing time is similar to that of the intermediate peak fraction, as shown in Fig. 5. On the other hand, we can simplify the phase mixing as random stacking based on the random-stacking model^28^. According to this model, the interface density could be calculated for our samples. As shown in Fig. 5, the profile of the interface density is similar to that of the SVF as well. Thus, we propose that the superconductivity in such a nanoscale system consisting of non-superconducting PI and PII phases can be associated with the interface between the metallic PI and the antiferromagnetic PII, which has been proposed to be an effective route to enhance or induce superconductivity^32,33^.

In conclusion, by carrying out low-temperature XRD and HRTEM measurements, we have determined the micro- and nano-scale structures and their evolution in low-temperature-annealed superconducting Ca122 samples. The experimental results are in excellent agreement with the NBD model. Our observations strongly support the conjecture that the superconductivity in undoped Ca122 is induced by interfaces between the PI and PII phases, although the detailed microscopic mechanism for the interface-induced superconductivity in Ca122 is yet to be determined.

## Methods

### Synthesis and preparation

The starting high-quality Ca122 single crystals were grown by the self-flux method^34^. First, the Fe and As powder were well mixed in an Ar-filled glovebox and sealed in an evacuated quartz tube that was heated at a temperature of 700 °C to obtain the FeAs precursor. The FeAs precursor and a Ca lump, in a molar ratio of 4:1, were then placed in an alumina crucible and sealed in a quartz tube under vacuum of 10^−6^ torr. The sealed tube was soaked at 1180 °C for 24 hours (h), and then gradually cooled down to 960 °C at a rate of 2 °C/h, followed by a furnace cooling down to room temperature. The pure PI phase was obtained by heating the re-sealed Ca122 sample to 850 °C and rapidly quenching to room temperature. Through subsequent annealing at 350 °C for different time periods, we obtained a series of Ca122 samples from pure PI phase to pure PII phase and in-between.

### Characterization

#### Physical property measurements

The magnetic properties (Fig. S1) were determined using the Quantum Design Magnetic Property Measurement System (MPMS).

#### X-ray diffraction

The structure at both room temperature (Fig. S2) and low temperature was determined by a Rigaku D-Max IIIB X-ray diffractometer equipped with an Oxford continuous flow cryostat using Cu K_α_ radiation. Each sample was cooled down slowly to a stable target temperature. After the temperature was stabilized, a θ/2θ reflection scan for the 2θ angle from 62° to 72° was carried out for the (008)-line to investigate the microstructural properties of Ca122.

#### Microscopy

Each as-prepared Ca122 specimen was cut into a rectangular slice (2 mm × 2 mm). We then exfoliated the slice along its c-axis with Scotch tape until its thickness reached around 10 μm. Subsequently, the sample (2 mm × 2 mm × 10 μm) was glued between two glass slide pieces (2 mm × 2 mm × 1 mm each) using G1 epoxy (Gatan). The sandwiched structure specimen was mechanically polished to about 100 μm in thickness using silicon carbide sandpaper (grit No. 2500), and then diamond paste with a particle size of 3 μm was used for final polishing. The polished sample was then bonded to a 3-mm-diameter molybdenum grid with a single hole of about 1 mm in diameter. Both sides of the sandwich specimen were dimpled using a dimple grinder (Model 656, Gatan), and the thinnest area was about 10 μm. The dimpled specimen was then ion-milled using a precision ion-polishing system (PIPS, Model 695, Gatan) with a liquid-nitrogen-cooled stage at 2 kV. For the HRTEM study, such prepared cross-section samples of both non-superconducting and superconducting Ca122 specimens were studied using an aberration-corrected JEM-ARM200CF microscope.

### Simulation

#### NBD model

We simulated the evolution of phase distribution for different annealing time periods by using a NBD toy model^30^. The cross section (*ac* plane) of the single crystal is represented by a 100 × 100 square matrix, where the PI and PII phases can be distinguished by different colors [red: PI and blue: PII in Fig. 4(a–c)]. Additionally, we simply assume that the matrix transforms linearly from PI- to PII-phase at a stable rate in order to dynamically simulate the experimental situations. During the simulation process, a time period of 0.1 seconds is chosen to stand for the time-length of a step. At each step, one square is randomly selected as a seed, and a random number is then generated to represent how many local squares around the seed will be filled. The random numbers are directly generated by the NBD, for which the expression for probability distribution is:

$$f({\rm{x}}|{\rm{r}},{\rm{p}})=\frac{{\rm{\Gamma }}({\rm{x}}-1+{\rm{r}})}{({\rm{x}}-1)!{\rm{\Gamma }}({\rm{r}})}{{\rm{p}}}^{{\rm{x}}}{(1-{\rm{p}})}^{{\rm{r}}},\,{\rm{for}}\,{\rm{x}}=1,2,3\,\ldots ,$$

where *p* represents the phase fraction of PII in the sample (in other words, *p* is directly related to the annealing time starting from the pure-PI phase) and *r* is a NBD parameter that controls the size of aggregation. Figure 4(a–c) visualize the simulations of possible structures with different *r*s. We chose the 15-h-annealed sample (15 seconds in simulation) as an example without loss of generality, at which the ratio of PI- to PII-phase is 1:1.

#### Intergrowth model

We employed an intergrowth model^31^ to simulate the XRD spectra with phase distributions generated using NBD discussed above, by which the XRD intensity **I**(**θ**) can be simulated with the structure, as shown in the following relation:

$${\rm{I}}({\rm{\theta }})={\rm{F}}|{\sum }_{n=1}^{m}{e}^{i{S}_{n}}{|}^{2},$$

with

$${{\rm{S}}}_{n}=\frac{4\pi \,\sin (\theta )}{\lambda }[{c}_{n}{d}_{PI}+(n-{c}_{n}){d}_{PII}],$$

where F is the structural factor (here we simplify the equation with F = 1); d_PI_ and d_PII_ are lattice parameters in *c*-axis that at 10 K are 10.72 Å and 11.55 Å, respectively; and c_n_ represents the layer number of PI phase started from the top layer to the nth layer.

## Supplementary information

Supplementary Information for

## Data Availability

The datasets generated during and/or analysed during the current study are available from the corresponding author on reasonable request.
