# Supplementary material for: Low-temperature microstructural studies on superconducting CaFe2As2
Source: Sci Rep. 2019 Apr 23;9:6393. doi: 10.1038/s41598-019-42660-6 (PMC6478709; doi:10.1038/s41598-019-42660-6)
Supplement: Supplementary file 1 — Supplementary Information for [file 41598_2019_42660_MOESM1_ESM.pdf]

Supplementary Information

Low-temperature microstructural studies on superconducting $CaFe_2As_2$

S. Huyan[1,*], L. Z. Deng[1], Z. Wu[1], K. Zhao[1], J. Y. Sun[1], L. J. Wu[2], Y. Y. Zhao[3], H. M. Yuan[1], M. Gooch[1], B. Lv[4], Y. Zhu[2], S. Chen[1] and C. W. Chu[1,5,†]

[1]Department of Physics and Texas Center for Superconductivity, University of Houston, Houston, TX 77204-5005
[2] Condensed Matter Physics and Materials Science Department, Brookhaven National Laboratory, Upton, NY 11973
[3] School of Physics and Optoelectronic Engineering, Nanjing University of Information Science and Technology, Nanjing, China
[4] Department of Physics, University of Texas at Dallas, Richardson, TX 75080
[5] Lawrence Berkeley National Laboratory, Berkeley, CA 94720
* shuyan@uh.edu
† cwchu@uh.edu

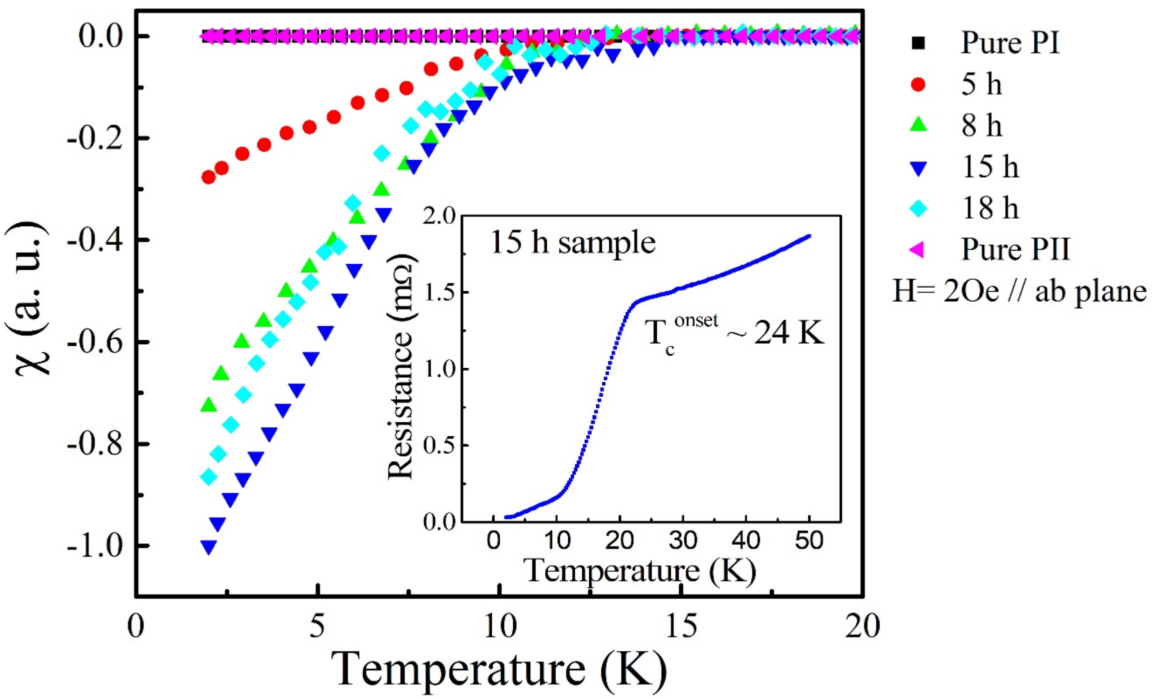

Figure S1. Magnetic susceptibility of undoped Ca122 samples annealed for different time periods measured under H = 2 Oe applied in the ab-plane direction through warming. Starting from the pure PI phase, the diamagnetic transition appears and then disappears as the PII phase becomes dominant with increasing annealing time. This indicates the induction and suppression of superconductivity in the samples. It is worth mentioning that the maximum shielding fraction is lower than 1%, which indicates the non-bulk nature of the observed superconductivity. Inset: Resistance-temperature curve of the sample annealed for 15 h.

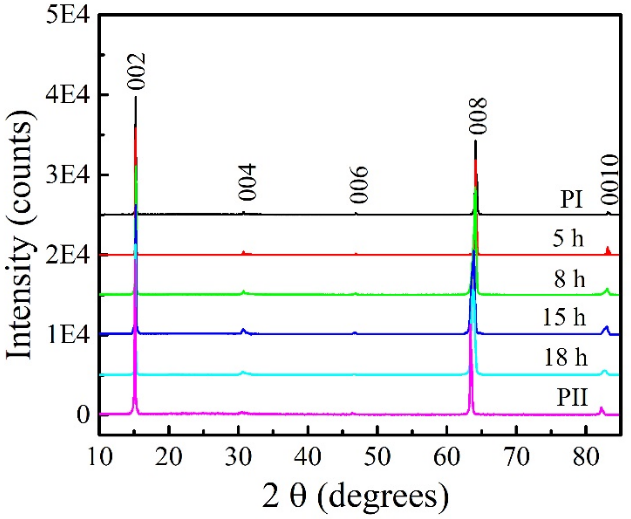

Figure S2. Room-temperature out-of-plane X-ray diffraction patterns of samples annealed for different time periods. Only the (00*l*) peaks with even *l* are evident, suggesting that the measured samples are well-oriented crystals. The shapes of the diffraction peaks are sharp for the pure-phase samples, while those for the mixing-phase samples are broad and asymmetrical. The systematic peak shift with different annealing time is exhibited.
